# Knowledge, Attitudes, Practices and Emotional Reactions among Residents of Avian Influenza (H5N1) Hit Communities in Vietnam

**DOI:** 10.1371/journal.pone.0047560

**Published:** 2012-10-19

**Authors:** Toshie Manabe, Tran Thuy Hanh, Doan Manh Lam, Do Thi Hong Van, Pham Thi Phuong Thuy, Dinh Thi Thanh Huyen, Tran Thi Mai Phuong, Dang Hung Minh, Jin Takasaki, Ngo Quy Chau, Ly Quoc Toan, Koichiro Kudo

**Affiliations:** 1 National Center for Global Health and Medicine, Tokyo, Japan; 2 Bach Mai Hospital, Hanoi, Vietnam; 3 Bac Kan General Provincial Hospital, Bac Kan Province, Vietnam; 4 NCGM-BMH Medical Collaboration Center, Hanoi, Vietnam; INSERM & Universite Pierre et Marie Curie, France

## Abstract

**Background:**

Awareness of individuals’ knowledge and predicting their behavior and emotional reactions is crucial when evaluating clinical preparedness for influenza pandemics with a highly pathogenic virus. Knowledge, attitude, and practice (KAP) relating to avian influenza (H5N1) virus infection among residents in communities where H5N1 patients occurred in Vietnam has not been reported.

**Methods and Principal Findings:**

Face-to-face interviews including KAP survey were conducted in Bac Kan province, located in the northeast mountainous region of Vietnam. Participants were residents who lived in a community where H5N1 cases have ever been reported (event group, n = 322) or one where cases have not been reported (non-event group, n = 221). Data on emotional reactions of participants and healthcare-seeking behavior after the event in neighboring areas were collected as well as information on demographics and environmental measures, information sources, and KAP regarding H5N1. These data were compared between two groups. Higher environmental risk of H5N1 and improper poultry-handling behaviors were identified in the event group. At the time of the event, over 50% of the event group sought healthcare for flu-like symptoms or because they were scared. Awareness of the event influenced KAP scores. Healthcare-seeking behavior and attention to H5N1 poultry outbreaks diminished in the event group as time passed after the outbreak compared with the non-event group. Factors that motivated participants to seek healthcare sooner were knowledge of early access to healthcare and the risk of eating sick/dead poultry, and perception of the threat of H5N1.

**Conclusions:**

Awareness of H5N1 patients in neighboring areas can provoke panic in residents and influence their healthcare-seeking behavior. Periodic education to share experiences on the occurrence of H5N1 patients and provide accurate information may help prevent panic and infection and reduce mortality. Local conditions should be taken into account when emphasizing the need for early access to healthcare.

## Introduction

Avian influenza A(H5N1) virus infection in humans remains rare and sporadic; however, it presents a continuous global pandemic threat associated with high mortality [1]. H5N1 infection is considered a very serious disease, especially among people living in high risk countries [2]. H5N1 infection can rapidly lead to severe pneumonia, acute respiratory distress syndrome (ARDS), and death [3]. Early initiation of antiviral treatment is recommended to treat H5N1 patients [4], [5] and the timing of such treatment is vital in effecting a positive outcome [6]. In cases of influenza A(H1N1)pdm09 virus infection, despite low lethality globally, there have been large numbers of hospitalized patients with acute and severe illness, and fatalities have occurred world-wide [7], [8], [9], [10], [11]. A study in Mexico indicated that early initiation of antiviral treatment can reduce the occurrence and severity of pneumonia including ARDS [12]. A study in Canada also showed that delayed antiviral treatment is independently associated with disease severity due to influenza A(H1N1)pdm09 virus infection [13]. Thus, regardless of the type of virus, early initiation of antiviral treatment is crucial in treating critically ill patients with influenza virus infection. However, early initiation of antiviral treatment requires early healthcare-seeking behavior on the part of the patient, and physicians who quickly initiate antiviral treatment after symptom onset [14].

Two laboratory-confirmed cases of H5N1 in humans were reported from a community in Bac Kan province, a mountainous region in northeastern Vietnam, in spring 2010 [15] during the H5N1 poultry outbreaks [16]. These two cases fortunately recovered. In one of these a successful outcome resulted from the short time (2 days) between symptom onset and hospitalization. Studies in previous years in Vietnam have reported elapsed time from symptom onset to hospitalization as several days [3], [17], [18], [19]. We hypothesized that the awareness of H5N1 patients would influence the knowledge and behavioral patterns of people who live in high-risk communities in Vietnam. Understanding of emotional reactions of H5N1 patients at the time of an occurrence can contribute to clinical preparedness for further highly pathogenic influenza pandemics. We conducted a face-to-face knowledge, attitude, and practice (KAP) survey regarding H5N1 in two communities in Bac Kan province, Vietnam, one with H5N1 patients in 2010 (event group) and one without patients (non-event group). The aim of the present study was to assess KAP and emotional reactions to H5N1 to facilitate the development of effective prevention and treatment strategies for H5N1 infection and further influenza pandemic, including early diagnosis and initiation of treatment.

## Materials and Methods

### Study Sites and Subjects

**Figure 1:**
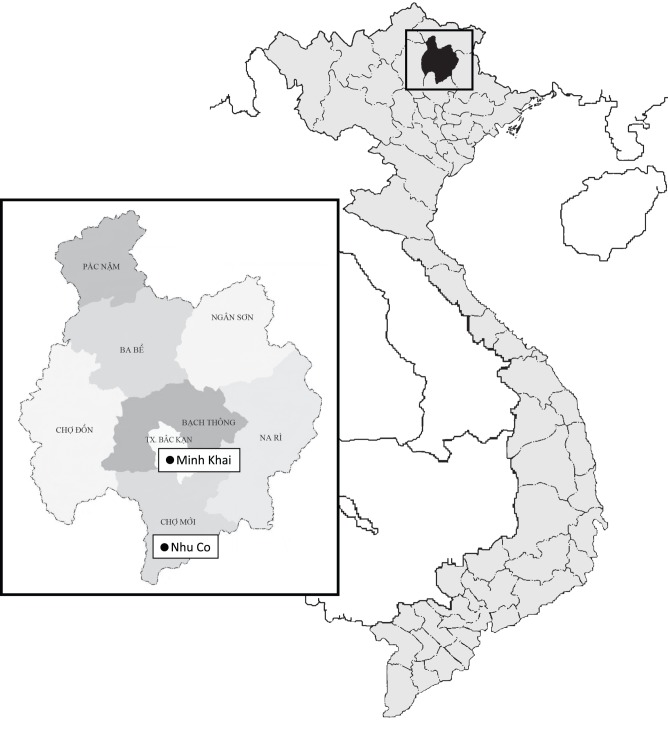
Locations of study sites. Bac Kan province is located in the mountainous region of northeast Vietnam. The white boxes denote study sites.

The study was performed in two communities: Nhu Co Commune in Cho Moi District and Minh Khai Ward in Bac Kan Township in Bac Kan province (Figure 1). The numbers of residents and households at each site were approximately 2,800 and 630, respectively, in Nhu Co and 4,600 and 750, respectively, in Minh Kai. Nhu Co is located in a more mountainous area of Bac Kan province than Minh Kai, which is located in the central town. At the time of the study, the average annual incomes per capita in Nhu Co and Minh Khai were $320 (US) and $680 (US), respectively. Bac Kan province has reported frequent H5N1 epizootic outbreaks among birds and domesticated poultry, including a report from Nhu Co Commune, Cho Moi District in spring 2010 [11]. Two H5N1 human cases were also reported in Cho Moi District between March and April 2010 [10]. The distance between the two study communities is approximately one hour by motorbike. There were commune health centers (CHC) in each study site to provide primary care services to residents including medical doctor and nurse.

The study investigators included healthcare providers at Bac Kan General Provincial Hospital who worked closely with the health department in Bac Kan province. The population list of study communities were maintained by that health department and obtained by the investigators. From these lists, residents younger than age 18 and older than age 70 were excluded. A total of 400 participants 18–70 years of age were randomly selected from the reminder on the population lists in Nhu Co (event group) and Minh Khai (non-event group) using a random number generator. The sample size was chosen to allow the detection of an effect of 0.25 with a power of 0.8, even after a substantial number of dropouts.

### Survey Methodology

The KAP survey was conducted in November 2011. The study subjects were invited to the survey by the letter from investigators and subjects who agreed to participate the survey received the face-to-face interview. The questionnaire was designed to assess KAP relating to H5N1 infection and emotional reactions to H5N1, and was back-translated. It collected information on the general background of participants, life environment in relation to household poultry, information sources on avian influenza, general knowledge of H5N1 infection, hygiene, and poultry handling, as well as emotional reactions to H5N1, including healthcare-seeking behaviors. The pilot survey was conducted at the Xuat Hoa Commune in Bac Kan Township, approximately one hour distance by motorbike from each study site, in September 2011, to identify suitable questions and interview methods for communities in Bac Kan province. All questions were either closed-ended or multiple-choice. The questionnaire was collected during face-to-face interviews conducted by previously-trained local healthcare workers. Interviews were held three times on a single day at meeting halls at each study site to avoid exchange of information among participants regarding the contents of the interviews. KAP associated with H5N1 infection were compared between the groups. A knowledge score was calculated according to correct answers. An attitude-practice score was also calculated to evaluate the factors influencing each score, including individuals’ healthcare behaviors. Economic conditions were classified according to quintiles of family income and were qualified on the basis of the possession of assets such as a television, radio, telephone, water server, refrigerator, buffalo/cow, bicycle, motorbike, car and air conditioner. Household poultry was defined as domesticated birds raised in backyards such as chickens, ducks, and musk ducks for the purpose of meet and eggs for daily meals and/or selling.

### Ethics

Ethical approval was provided by the Institutional Review Board of the Ministry of Health, Vietnam, Bach Mai Hospital and the National Center for Global Health and Medicine, Japan. All study participants provided either written informed consent or verbal consent if they were illiterate. This method was approved for the present study in Vietnam by ethical review boards. Consent was documented with the participants' signature or figure prints if they were illiterate, according to rules for scientific research in Vietnam.

### Statistical Analysis

Survey data were double-entered and analyzed using SPSS Statistics ver. 20 (IBM, Armonk, NY, USA). Continuous variables were compared using Mann-Whitney U or Kruskal-Wallis tests. Categorical variables were analyzed using chi-square tests and Fisher’s exact tests. A maximum of three points was assigned to the answer ‘agree’ for each question, two points were assigned to ‘undecided,’ and one point to ‘disagree,’ according to a three-point Likert-type scale. KAP scores were calculated according to the answers using factor analysis, adjusted to give a total score of 10. Factors influencing KAP scores were analyzed by logistic regression analysis. Independent factors influencing early access to healthcare were analyzed using a step-wise selection method to select variables from the baseline backgrounds of participants. For all analyses, significance levels were two-tailed, and a P value of <0.05 was considered significant.

## Results

### General Background of Study Participants

**Figure 2:**
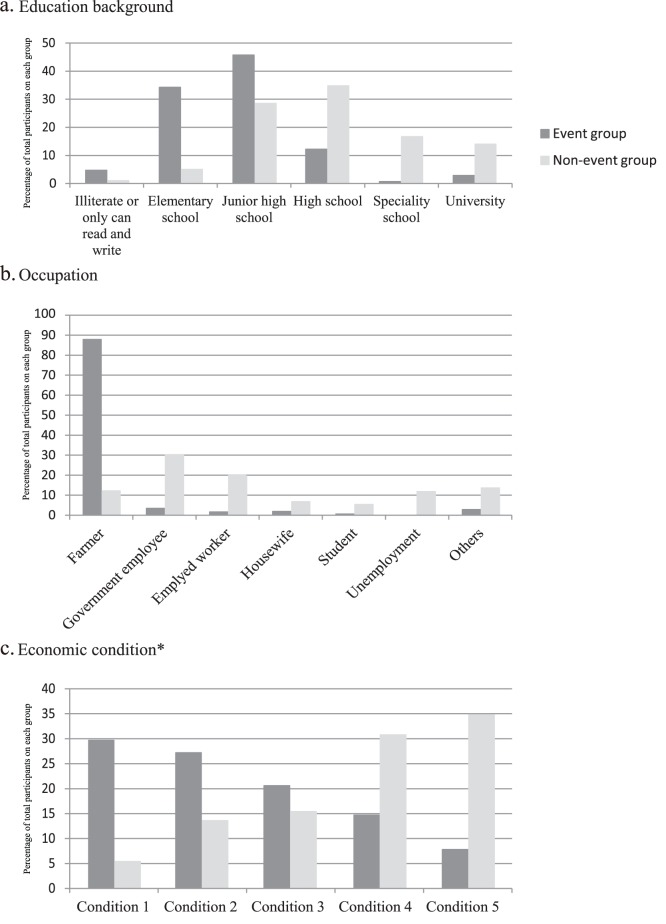
General backgrounds of study participants. Backgrounds on education (a), occupation (b), and economic condition (c) of study participants are compared between the event group and the non-event group. *Economic condition was qualified based on the possession of assets such as a television, radio, telephone, water server, refrigerator, buffalo, bicycle, motorbike, car and air conditioner, and was divided into quintiles according to family income.

Totals of 322 (median age, 36 [IQR 26–47] years) and 221 (median age, 40 [IQR 32–52] years) participants from the event group and non-event group, respectively, agreed to participate in the present study. The general backgrounds of the participants are shown in Table 1 and Figure 2. The educational level was lower in participants in the event group, which included 4.7% with no educational background, compared with 1% in the non-event group (P<0.001). There was a significant difference in occupations between the groups (P<0.001), with most participants in the event group being farmers (87.9%), compared with government employees (30.3%) and employed workers (19.9%) in the non-event group. Participants in the non-event group also had a significantly higher economic condition (≥condition 4) compared with the event group (P<0.001). More than 80% of participants overall had health insurance, but significantly more participants in the event group had insurance compared with the non-event group (P<0.014).

**Table 1:** Background of study participants.

	Event group (Nhu Co)	Non-event group (Minh Kai)	P value
	n = 322	n = 221	
	No. (%)	No. (%)	
**General characteristics of subjects**			
Gender - male (%)	103 (32.0)	72 (32.6)	0.926
Age –median (IQR) yr.	36.0 (26–47)	40.0 (32–52)	0.001
Education, No. (%)			<0.001
Illiterate or only can read and write	15 (4.7)	2 (1.0)	
Elementary school	110 (34.2)	11 (5.0)	
Junior high school	147 (45.7)	63 (28.5)	
High school	39 (12.1)	77 (34.8)	
Specialty school	2 (0.6)	37 (16.7)	
College/University	9 (2.8)	31 (14.0)	
Occupation			<0.001
Farmer	283 (87.9)	27 (12.2)	
Housewife	6 (1.9)	15 (6.8)	
Government employee	11 (3.4)	67 (30.3)	
Employed worker	5 (1.6)	44 (19.9)	
Student	2 (0.6)	12 (5.4)	
Unemployment	0 (0.0)	26 (11.8)	
Others	9 (2.8)	30 (13.6)	
Economic condition*			<0.001
1	95 (29.7)	12 (5.4)	
2	87 (27.2)	30 (13.6)	
3	66 (20.6)	34 (15.4)	
4	47 (14.7)	68 (30.8)	
5	25 (7.8)	77 (34.8)	
Health insurance	290 (90.1)	183 (82.8)	0.014
**Life environment**			
Number of participant owing household poultry	298 (92.5)	112 (50.7)	<0.001
Number of poultry raised at the participants’ house			
Chickens			<0.001
<10	43 (13.4)	42 (19.0)	
≥10	270 83.8)	70 (31.7)	
Ducks			<0.001
<10	19 (5.9)	1 (0.5)	
≥10	50 (15.6)	3 (1.4)	
Musk ducks			<0.001
<10	21 (6.5)	3 (1.4)	
≥10	56 (17.4)	0 (0.0)	
**How often do you have contact with poultry?**			<0.001
Frequently	264 (82.0)	91 (41.2)	
Sometimes	53 (16.5)	118 (53.4)	
**Have your poultry been vaccinated against AI?**	276 (85.7)	174 (78.7)	0.037
**How do you protect backyard poultry form AI infection**			
Vaccination	276 (85.7)	174 (78.7)	0.037
Clean or disinfect poultry cage	150 (46.6)	130 (58.8)	0.005
Keep poultry in good condition	114 (35.4)	82 (37.1)	0.716
Built fence around the area	46 (14.3)	79 (35.7)	<0.001
Do nothing	10 (3.1)	3 (1.4)	0.257
**Backyard poultry ever infected with AI**	110 (34.2)	15 (6.8)	<0.001
**Backyard poultry ever experienced sudden death**	162 (50.3)	51 (23.1)	<0.001

* Economic condition was qualified based on the possession of assets such as a television, radio, telephone, water server, refrigerator, buffalo, bicycle, motorbike, car and air conditioner, and was divided into quintiles according to family income. AI: avian influenza (H5N1) virus infection in humans.

Participants who possessed household poultry were more common in the event group (92.5%) than in the non-event group (50.7%). Furthermore, significantly more participants in the event group had contact with poultry as part of their daily work (P<0.001). Significantly more participants in the event group had experienced H5N1 infection and sudden death in household poultry compared with the non-event group (P<0.001).

### Information Sources and their Effects on Behavior in Relation to H5N1

Most participants had heard about avian influenza (H5N1) (event group 97.2%, non-event group 98.2%, P = 0.263). Sources of their information relating to avian influenza (H5N1) are listed in Table 2. The most common information source for participants in both groups was the television (event group 73.9%, non-event group 91.4%, P<0.001) followed by radio (event group 59.3%, non-event group 48.0%, P = 0.011). Newspapers were not identified as a major source of information, especially in the event group.

**Table 2:** Information sources and reactions to knowing there were H5N1 patients in neighboring areas.

	Event group (Nhu Co)	Non-event group (Minh Kai)	P value
	(n = 322)	(n = 221)	
	No. (%)	No. (%)	
**Ever heard about AI**	313 (97.2)	217 (98.2)	0.263
**Information**			
Information source			
Television	238 (73.9)	202 (91.4)	<0.001
Radio	191 (59.3)	106 (48.0)	0.011
Newspaper	13 (4.0)	61 (27.6)	<0.001
Poster	2 (0.6)	25 (11.3)	<0.001
Friend	29 (9.0)	30 (13.6)	0.122
Healthcare worker	100 (31.1)	77 (34.8)	0.402
Advertisement of women’s association	82 (25.5)	79 (35.7)	0.013
Others	3 (0.9)	7 (3.2)	0.099
Ever attended any educational programs relating to H5N1?	216 (67.1)	161 (72.9)	0.130
Requested more information on AI	303 (94.1)	213 (96.4)	0.262
**News about the occurrence of H5N1 patients in Cho Moi District in 2010**			
Know about the news*	280 (87.0)	168 (76.0)	0.001
Scared if get AI	287 (89.1)	160 (72.4)	<0.001
Visited hospital with some symptoms	174 (54.0)	87 (39.4)	0.001
Kind of symptoms that you got			
Fever	149 (46.3)	61 (27.6)	<0.001
Dyspnea	33 (10.2)	10 (4.5)	0.015
Cough	145 (45.0)	48 (21.7)	<0.001
Sneezing	39 (12.1)	39 (17.6)	0.081
Nasal discharge	54 (16.8)	43 (19.5)	0.427
Just scared	17 (5.3)	9 (4.1)	0.548
**After the news** *			
Pay more attention to dead/sick poultry	297 (92.2)	213 (96.4)	0.066
Seek healthcare earlier once get symptoms	293 (91.0)	220 (99.5)	<0.001

* Occurrence of H5N1 patients in Cho Moi District, Bac Kan province in the spring of 2010. AI: avian influenza (H5N1) virus infection in humans.

More participants in the event group knew about the occurrence of H5N1 patients in Cho Moi District in the spring of 2010 (event) compared with participants in the non-event group (P = 0.001) (Table 2). Participants from both groups who knew about the event were scared when they heard the news. Upon receiving the news, 54.0% of participants in the event group and 39.4% in the non-event group went to the hospital with symptoms of fever, cough, sneezing, nasal discharge, or just because they were scared. After the event, significantly more participants in the non-event group tended to seek healthcare earlier when they developed symptoms, compared with the event group (P<0.001).

### KAP of Study Participants Related to Avian Influenza (H5N1)

Some of the KAP results are shown in Tables 3 and 4. Although participants in both groups displayed a high level of knowledge, the non-event group provided significantly more correct answers to questions related to whether H5N1 was an infectious disease, its mode of transmission, whether it can be prevented or cured, and the likelihood of dying. Increased awareness about the importance of early access to healthcare for treating H5N1 was assessed, and approximately half of the participants in the event group were unaware that they should not eat poultry that had become ill or died for unknown reasons, compared with over 70% of participants in the non-event group (P<0.001). Study participants in both groups listed avian influenza (H5N1) infection as the most serious illness concerning them.

**Table 3:** Knowledge of study participants regarding avian influenza (H5N1).

	Event group (Nhu Co)	Non-event group (Minh Kai)	P value
	(n = 322)	(n = 221)	
	No. (%)	No. (%)	
AI^†^ is a kind of infectious disease	303 (94.1)	216 (97.7)	0.042
People get AI by touching sick poultry	293 (91.0)	214 (96.8)	0.003
AI can be prevented	292 (90.7)	214 (96.8)	0.001
AI can be cured	276 (85.7)	198 (89.6)	0.001
People can die of AI	298 (92.5)	214 (96.8)	0.021
Do not eat sick/dead poultry	165 (51.2)	158 (71.5)	<0.001
Early access to healthcare is the key to treat AI	312 (96.9)	215 (97.3)	0.805
What is the most serious disease that you concern			
Diarrhea	57 (17.7)	38 (17.2)	0.909
Cough/pneumonia	38 (11.8)	18 (8.1)	0.197
Avian influenza (H5N1)	236 (73.3)	157 (71.0)	0.625
Malaria	50 (15.5)	19 (8.6)	0.018
Tuberculosis	52 (16.1)	20 (9.0)	0.020
Others	10 (3.1)	11 (5.0)	0.003

AI: avian influenza (H5N1) in humans.

**Table 4:** Attitude-practices related to avian influenza (H5N1).

	Event group (Nhu Co)	Non-event group (Minh Kai)	P value
	n = 322	n = 221	
	No. (%)	No. (%)	
**Hygiene practices**			
Use soap when you wash your hands	318 (98.8)	220 (99.5)	0.766
Use clean water when you wash your hands	245 (76.1)	208 (94.1)	<0.001
What would you do if your household poultry suddenly die? (multiple choices)*			
Sell rest of live poultry	10 (3.1)	1 (0.5)	0.032
Eat them	3 (0.9)	1 (0.5)	0.649
Throw them in a river or pond/outside	6 (1.9)	2 (0.9)	0.482
Bury them	301 (93.5)	177 (80.1)	<0.001
Disinfect poultry cage	137 (42.5)	101 (45.7)	
Report to government authorities	173 (53.7)	149 (57.4)	0.002
Do nothing	2 (0.6)	0 (0.0)	0.516
When slaughtering poultry, how can you protect yourself from AI? (multiple choices)			
Wear gloves	155 (48.1)	142 (64.3)	<0.001
Wear mask	159 (49.4)	123 (55.7)	0.162
Do it away from house	40 (12.4)	60 (27.1)	<0.001
Wash hands afterwards with soap	279 (86.6)	195 (88.2)	0.603
Clean area afterwards…	110 (34.2)	160 (72.4)	<0.001
**Healthcare-seeking behavior**			
After touching sick or dead poultry, if you are sick with fever, how fast do you seek treatment?			0.002
Immediately	250 (77.6)	200 (90.5)	
1–2 days after onset	55 (17.1)	16 (7.2)	
If get really sick	11 (6.4)	3 (1.4)	
Nothing	1 (0.3)	1 (0.5)	
Which organization do you seek treatment at first?			
Community health center	291 (90.4)	56 (25.3)	<0.001
District hospital	52 (16.1)	30 (13.6)	0.465
Provincial hospital	2 (0.6)	137 (62.0)	<0.001

AI: avian influenza (H5N1) virus infection in humans.

* participants who did not have household poultry answered as if they have household poultry.

Many participants in both groups said that they buried household poultry if that died suddenly, but some participants in the event group said that they sold the remainder of the live poultry (3.1%), ate the dead poultry (0.9%), or threw the carcasses into rivers or ponds (1.9%). When they slaughtered their poultry, significantly fewer participants in the event group compared with the non-event group said that they wore gloves (p<0.001) and did it away from their house (p<0.001).

Significantly fewer participants in the event group compared with the non-event group said that they sought healthcare immediately if they developed a fever after touching sick poultry (P<0.001). The preferred healthcare organization that they accessed first when they got a fever was the commune healthcare center in the event group (90.4%) and the provincial hospital in the non-event group (62.0%).

The median knowledge and attitude-practice scores in the event and non-event groups were 8 vs. 9, and 6.9 vs. 7.4, respectively, out of a total of 10. The differences between the groups in terms of both scores were significant (P<0.001) (Table 5).

**Table 5:** Knowledge and attitude-practices scores.

	Median	Range	Z*	P value
**Knowledge score**				
Event group (Nhu Co)(n = 322)	8	1–10	−4.542	<0.001
Non-event group (Minh Kai) (n = 221)	9	2–13		
**Attitude-practice score**				
Event group (Nhu Co)(n = 322)	6.9	3–10	−6.482	<0.001
Non-event group (Minh Kai) (n = 221)	7.4	4–9		

* The Z statistic was obtained from the Mann-Whitney test for two independent samples.

### Factors Influencing Early Access to Health Care

In the logistic regression analysis using baseline background data of the participants, factors that influenced early access to healthcare once participants developed symptoms were knowledge about the necessity of early access to healthcare for treating avian influenza, not eating sick and dead poultry, and considering avian influenza to be a life-threatening disease (Table 6).

**Table 6:** Factors influencing early access to healthcare identified by logistic regression analysis.

	Coefficient	Standard error	P value	Odds ratio	95% confident interval
Constant	0.382	0.735	0.603	1.465	
Know that early access to healthcare is the keyto treating AI*	2.000	0.740	0.007	7.390	1.731–31.529
Know not to eat sick/dead poultry	1.146	0.557	0.040	3.145	1.056–9.367
AI is the most life-threatening disease	1.233	0.526	0.019	3.433	1.225–9.624

AI: avian influenza (H5N1) virus infection in humans.

## Discussion

The present study demonstrated that the occurrence of H5N1 in neighboring areas had an emotional impact, and also increased people’s attention to preventive measures and their knowledge about the necessity of early access to healthcare. Information and education delivery need to take into account the local conditions of the population.

Most human cases of avian influenza (H5N1) occur through direct or indirect contact with poultry [20], [21], [22]. Contaminated water has also been identified as a potential risk factor [23], [24]. Bac Kan province is in a mountainous part of northeast Vietnam, with 95% of its area dominated by forest. The event group in Cho Moi District was located in a more deeply-forested area than the non-event group even though the districts are located side-by-side in Bac Kan province (Figure 1). Along with the study participants, most residents in Cho Moi district were farmers and raised chickens, ducks, buffalo, and pigs. The non-event group lived in a more urban environment. Even though high awareness of H5N1 infection was observed in both groups, the event group had more chance to come into contact with poultry, thus increasing their risk for H5N1 infection compared with the non-event group. These results were compatible with a previous study in China that compared a typical urban area with a rural area in the middle of China [25]. More participants in the event group had experienced H5N1 infection and unexplained sudden death in their household poultry, and a higher awareness of the importance of vaccination of poultry was observed. However, when their poultry died suddenly, participants in the event group were more likely than those in the non-event group to sell the rest of their live poultry, eat dead poultry, or throw carcasses into a river or pond. These results indicate that, participants in the event group maintained traditional habits and did not sufficient knowledge about the risks of H5N1 infection, with economic difficulties possibly contributing factor. Their economic difficulties in the event group might contribute for these behaviors. Although the role of water in transmission could not be confirmed, people’s behavior can contribute to the production of contaminated water. The results also indicated a lower knowledge score in the event group than in the non-event group. The main information sources about H5N1 infection for both groups were the television and radio. However, differences in economic conditions, which were qualified on the basis of household possessions, explained the difficulty in receiving information from television and radio, given that most participants in the event group did not have access to these. Educational background was also an important factor in understanding the information. Over 34% of participants in the event group had only an elementary school education, were illiterate, or had no educational background. Increased knowledge and appropriate attitude-practices relating to H5N1 infection were influenced by education, occupation, and economic conditions among the study population in an H5N1-affected community, as well as by the awareness of the presence of H5N1 patients in the community. These findings are compatible with previous surveys relating to H5N1 in China, Afghanistan, Laos, and Italy [25], [26], [27], [28], and suggest that appropriate information delivery needs to be adjusted to local conditions [2], [25].

More participants in the event group knew about the occurrence of H5N1 patients in neighboring areas (P = 0.001) and were scared when they heard the news of an event (P<0.001). At the time of the event, 174 (54%) of participants in the event group and 168 (76%) of participants in the non-event group visited the hospital with flu-like symptoms such as cough, fever, and nasal discharge, or just because they were scared. The occurrence of H5N1 infection in humans in their neighbors caused the residents to panic and be unable to make calm decisions. They were unsure if their symptoms were attributable to H5N1 or to infection by another influenza virus, and their resulting behaviors made it more difficult for medical providers to take care of those who really needed medical intervention. After the event, almost all participants in the non-event group and over 90% in the event group sought healthcare early once they developed symptoms (e.g., fever). The event thus had an impact on their healthcare-seeking behaviors.

Logistic regression analysis identified factors influencing immediate access to healthcare once participants developed a fever after touching sick/dead poultry as knowledge about not eating such poultry, knowledge about the necessity for early access to healthcare, and recognition of H5N1 as a life-threatening disease. The results indicate that healthcare providers in high-risk areas need to stress the necessity of early access to healthcare, and promote proper knowledge about poultry handling to prohibit habits that favor H5N1 infection. Participants in the event group visited their local health center, while participants in the non-event group visited the provincial hospital. It is difficult to change behaviors and customs, especially in residents of rural areas. However, closer relationships between local healthcare providers and residents could promote early healthcare behaviors in people living in rural communities in deeply-forested regions. Educational programs conducted by local healthcare providers might be effective, but the attitudes of local residents must be taken into consideration when planning health education in communities with H5N1 patients in neighboring areas.

This study was limited to a comparison of participants living in one community affected and the other unaffected by the H5N1 outbreak in 2010, representing a rural and an urban setting in a province of Vietnam. Participants who did not have household poultry were included in the study participants and they need to answer some questions as if they have household poultry. Further investigations comparing subjects with similar socioeconomic conditions and common educational and environmental backgrounds are required to further assess the influence of an H5N1 outbreak.

Awareness of H5N1 patients in neighboring areas can cause panic in residents. However, it can also contribute to early healthcare-seeking behavior. Providing information from experiences of occurrence of H5N1 patients and clinical preparedness are crucial if further influenza pandemics occurred. Periodic educational interventions using locally-adjusted methods could contribute to preventing panic, motivating early access to healthcare, and reducing infection and mortality.
